# Harnessing Potential of ω-3 Polyunsaturated Fatty Acid with Nanotechnology for Enhanced Breast Cancer Therapy: A Comprehensive Investigation into ALA-Based Liposomal PTX Delivery

**DOI:** 10.3390/pharmaceutics16070913

**Published:** 2024-07-09

**Authors:** Rohit Kumar, Anurag Kumar, Dharmendra Kumar, Sneha Yadav, Neeraj Kumar Shrivastava, Jyoti Singh, Archana Bharti Sonkar, Pratibha Verma, Dilip Kumar Arya, Gaurav Kaithwas, Ashish Kumar Agrawal, Sanjay Singh

**Affiliations:** 1 Department of Pharmaceutical Sciences, School of Pharmaceutical Sciences, Babasaheb Bhimrao Ambedkar University (A Central University), Vidya Vihar, Raebareli Road, Lucknow 226025, India; rohitpharm06@gmail.com (R.K.); anuragkumar173@gmail.com (A.K.); dkpharm.25@hotmail.com (D.K.); sneha1991yad@gmail.com (S.Y.); neerajsrivastava91@gmail.com (N.K.S.); jsjyoti156@gmail.com (J.S.); sonkararchana818283@gmail.com (A.B.S.); pratibhaverma2208@gmail.com (P.V.); dkaharsh101@gmail.com (D.K.A.); gauravk@bbau.ac.in (G.K.); 2 Department of Pharmaceutical Engineering and Technology, Indian Institute of Technology (BHU), Varanasi 221005, India; 3 Dr. Shakuntala Misra National Rehabilitation University, Mohaan Road, Lucknow 226017, India

**Keywords:** liposome, α-linolenic acid, paclitaxel, cell culture, cancer

## Abstract

Our hypothesis posited that incorporating alpha-linolenic acid (ALA) into liposomes containing Paclitaxel (PTX) could augment cellular uptake, decrease the therapeutic dosage, and alleviate PTX-related side effects. Our investigation encompassed characterization of the liposomal formulation, encompassing aspects like particle size, surface morphology, chemical structure, drug release kinetics, and stability. Compatibility studies were performed through Fourier transform infrared spectroscopy (FTIR). By utilizing the Box–Behnken design (BBD), we developed ALA-based liposomes with satisfactory particle size and entrapment efficiency. It is noteworthy that ALA incorporation led to a slight increase in particle size but did not notably affect drug entrapment. In vitro drug release assessments unveiled a sustained release pattern, with ALA-PTX liposomes demonstrating release profiles comparable to PTX liposomes. Morphological examinations confirmed the spherical structure of the liposomes, indicating that substituting ALA with phosphatidylcholine did not alter the physicochemical properties. Cellular uptake investigations showcased enhanced uptake of ALA-based liposomes in contrast to PTX liposomes, likely attributed to the heightened fluidity conferred by ALA. Efficacy against MCF-7 cells demonstrated concentration-dependent reductions in cell viability, with ALA-PTX liposomes exhibiting the lowest IC50 value. Morphological analysis confirmed apoptotic changes in cells treated with all formulations, with ALA-PTX liposomes eliciting more pronounced changes, indicative of enhanced anticancer efficacy.

## 1. Introduction

Cancer disrupts the normal process of cell death, leading to the uncontrolled growth of abnormal or damaged cells that form masses known as tumors [1]. Among women globally, breast cancer stands as the most prevalent solid tumor and ranks second in cancer-related female mortality following lung cancer. In India, a woman is diagnosed with breast cancer every four minutes, contributing to 10.4% of all cancer cases in Indian women [2]. Traditional treatments for breast cancer encompass surgery, radiation, chemotherapy, and hormonal therapy. However, these treatments often bring about various side effects, like lymphedema, hot flashes, bone density reduction, and neuropathy. Consequently, there exists a pressing need for innovative treatment modalities that offer heightened therapeutic effectiveness while minimizing side effects [3]. 

The protein–lipid–protein pattern of the cell membrane comprises three layers, with proteins accounting for 52%, lipids for 40%, and carbohydrates for 10% of the composition [4]. The organization and composition of membranes play a crucial role in cell survival, particularly in breast cancer. The elevated lipid content in the plasma membrane of cancer cells offers lipid flexibility, contributing to mechanical stability during cell division and reduced shear force during cell separation [5]. Additionally, cancer cells remodel their plasma membrane to promote proliferation, evade apoptosis, and resist anticancer drugs, a primary factor in multidrug resistance (MDR) in cancer therapy [6]. Alpha-linoleic acid (ALA), a polyunsaturated fatty acid (PUFA) derivative, plays a significant role in blood clotting, inflammation, blood vessel function, and normal brain and ocular retina function. Due to their favorable effects on health, dietary supplements containing ALA have gained popularity worldwide. ALA has been proposed for the treatment of various medical conditions, including cardiovascular disease, inflammation, and cancer [7]. PUFAs serve as crucial structural elements of cell membranes, regulating cellular fluidity, permeability, and signaling pathways upon incorporation into phospholipid membranes. Therefore, if PUFAs enhance plasma membrane permeability and fluidity, they can potentially facilitate the diffusion of anticancer agents with poor membrane permeability [8]. 

Paclitaxel (PTX) is recognized by the National Cancer Institute (NIC) as a microtubulin stabilizing agent. It exists as an off-white crystalline powder with low water solubility and membrane permeability, melting at approximately 216–217 °C. The half-life of PTX ranges from 1.3 to 8.6 h [9]. 

Previous research from our laboratory has highlighted the efficacy of alpha-linolenic acid (ALA) in combating oxidative stress [10]. We have also investigated ALA’s anticancer potential against mammary gland carcinoma induced by MNU and DMBA, particularly its effectiveness against ER + MCF-7 cells. Our findings indicated that ALA downregulates Fatty Acid Synthase (FASN), thereby promoting mitochondrial apoptosis [11]. Furthermore, our studies have shown that ALA can compete with arachidonic acid (AA), a pivotal PUFA in cell membranes, during inflammatory cascades, thereby dampening inflammatory signals. With the well-documented anticancer activity of ALA, particularly its ability to compete with AA, we deemed it valuable to design an ALA-based liposome containing PTX. Our hypothesis posited that ALA would enhance the cellular uptake of PTX and reduce the required dosage, thereby mitigating the associated side effects. It is worth noting that in our proposed work, we endeavored to replace other lipids (such as phosphatidylcholine and cholesterol) typically used in liposomal preparations with ALA. Hence, this study aims to develop ALA-loaded PTX liposomes and assess their anticancer potential. 

## 2. Material and Methods

### 2.1. Materials and Reagent

PTX was generously provided as a gift sample by Fresenius Kabi Oncology Limited in Gurugram, India. Alpha-linolenic acid (ALA) was extracted from *Linum usitatissimum* (Flaxseed/Linseed) within our laboratory, as detailed in the publication (https://doi.org/10.1016/j.lwt.2023.115466), published date: 25 October 2023. Cholesterol was procured from Loba Chemie Pvt. Ltd. in Mumbai, India. D-alpha-tocopheryl polyethylene glycol 1000 succinate (TPGS) was sourced from Antares Health Products in St. Charles, MO, USA, and phosphatidylcholine (PC) was obtained from VAV Lipids Pvt. Ltd., 51/B Mittal Court, Mumbai, India. The dialysis bag used had an 8–14 kDa molecular weight cut-off and was purchased from Sigma-Aldrich, Burlington, MA, USA. Chloroform and methanol were acquired from Finar, while all other chemicals were sourced from Genetix Biotech Asia Pvt. Ltd. in New Delhi, India, or as otherwise specified in the text. 

### 2.2. Pre-Formulation Study

We conducted pre-formulation studies to assess the physicochemical properties of the drug. Additionally, we determined the compatibility of PTX and ALA with excipients to prevent any potential incompatibilities (Table S1). 

### 2.3. Liposome Preparation and Optimization

#### 2.3.1. Design of Experiment

The optimization of ALA- and PTX-loaded liposomal formulations using Design Expert software-13 involves a systematic approach to fine-tuning various formulation parameters to achieve the desired outcomes. In this study, the Box–Behnken design was applied, which is a type of response surface methodology (RSM) that facilitates the exploration of quadratic response surfaces and is efficient in terms of the number of experimental runs required. PC (mg), CHOL (mg), and ALA (µL) were selected as independent variables and PS, PDI, and ZP as dependent variables. Through this design, interactions between the variables and their individual and combined effects on the responses were analyzed. The software application generated mathematical models and 3D response surface plots that illustrated how changes in PC, CHOL, and ALA affected the PS, PDI, and ZP of the liposomes. Using Design Expert software with the Box-Behnken design, the optimal formulation was determined using desirability function analysis targeting minimized particle size (<200 nm), optimized PDI (<0.4), and appropriate zeta potential for stability. The predicted optimal parameters were PC: 15 mg, CHOL: 10 mg, and ALA: 55 μL, yielding predicted responses of particle size 154.4 nm, PDI 0.25, and zeta potential −22 mV. The final experimental formulation used PC: 20 mg, CHOL: 10 mg, ALA: 60 μL, which was based on the maximum entrapment and loading efficiency observed with this ratio as compared to the optimized formulation. The optimal formulation was achieved through a systematic approach involving multiple stages of analysis and evaluation (Tables S2 and S3) [12]. 

#### 2.3.2. Preparation of Liposomes

The PTX-loaded ALA liposomes were prepared with minor modifications to the thin-film hydration method. Initially, phosphatidylcholine (PC), cholesterol (CHO), and D-alpha-tocopheryl polyethylene glycol 1000 succinate (TPGS) (20 mg:10 mg: 5 mg) were dissolved in a solvent mixture of chloroform and methanol (2:3 ratio) within a 10 mL round-bottom flask (RBF). PTX (10 mg) and ALA (60 µL) were also mixed in the organic solvent and then slowly evaporated in a rotary evaporator under vacuum pressure to form a thin lipid coating. Subsequently, the produced film underwent further drying in a vacuum oven set to 25 °C for 4 h to remove any residual organic solvent. The thin film was then hydrated for 2 h at 100 rpm by using milli-Q water. Finally, the liposomal dispersion was probe-sonicated (MiSonix, 625 East Bunker Court, Vernon Hills, IL, USA) for an optimized duration to achieve smaller and uniformly sized vesicles [13]. 

### 2.4. Characterization of Liposomes

#### 2.4.1. PS, PDI, and Zeta Potential

All formulations were assessed for PS, PDI, and Zeta potential by using Zeta sizer (Nano ZS; Malvern Instruments, Malvern, UK). 

#### 2.4.2. Compatibility Studies Using FTIR

The excipients and liposome samples were deposited onto KBr disks for FTIR spectroscopy using a Nicole 6700 spectrometer from Thermo Scientific, Waltham, MA, USA. Scanning was performed across the range of 400–4000 cm^−1^ at a speed of 2 mm/s and a resolution of 4 cm^−1^ at room temperature. The bandwidth was measured at 50% of the peak height for accurate analysis of the spectra [14]. 

#### 2.4.3. Entrapment Efficiency

The liposomes underwent centrifugation at 10,000 rpm for 10 min to release the free drug. The drug-containing supernatant was then collected and subjected to further centrifugation at 15,000 rpm for 40 min at 4 °C by using a Hitachi WX Series centrifuge to isolate the liposome pellet. These pellets were subsequently dispersed in acetonitrile, and the concentration of PTX was measured by using an optimized UV–visible spectroscopy method [15]. 

#### 2.4.4. In-Vitro Drug Release

In vitro drug release from liposomes was performed by using the dialysis bag method [16,17]. The dialysis bag was activated by immersing it in 50 mL of phosphate buffer solution (pH 7.4) for 24 h at 37 °C in a shaking water bath spinning at 100 rpm. Subsequently, 2 mL of liposomes was filled into the dialysis membrane, then membrane was tied securely at both ends. The membrane containing liposome was then immersed in a beaker containing 50 mL of phosphate buffer solution (pH 7.4) and placed on a digital magnetic stirrer set to 37 °C and 100 rpm. During the experiment, the release medium was periodically sampled and replaced with fresh medium at specific time intervals. The total cumulative amount of drug released from the liposomes was quantified by measuring the drug concentration at each sampling time by using UV–visible spectroscopy at 230 nm [18]. To evaluate the kinetics and mechanism of drug release, in vitro release data were also presented for the zero order, first order, Higuchi model, and Korsmeyer–Peppas model [19]. 

#### 2.4.5. Scanning Electron Microscopy

The liposome samples were affixed to scanning electron microscope (SEM) specimen stubs by using double-sided carbon tape. Subsequently, an Au–Pd sputter-coating process was performed for 70 s to enhance conductivity and imaging quality. The analysis was conducted by using SEM at various accelerating voltages and magnifications to examine the surface morphology and structure of the liposomes (JSM 6490 LV; JEOL, Tokyo, Japan) [20]. 

#### 2.4.6. Transmission Electron Microscopy

Transmission electron microscopy (TEM) was utilized to characterize the liposomes. The liposome suspensions were diluted with water and then applied onto a copper grid coated with Formvar. Subsequently, a phosphor tungstic acid solution was used to negatively stain the grid. The sample was air-dried at room temperature before being evaluated with TEM for a detailed examination of the liposome structure and morphology (H-7650; HITACHI, Tokyo, Japan) [21]. 

#### 2.4.7. Accelerated Stability Study

To assess the stability of the liposomes, an optimized batch of liposomal dispersion was stored in air-tight sealed vials at temperatures ranging from 8 °C to room temperature (37 °C). The samples were placed in stability ovens (DD-155H; Meta-Lab Scientific Industries, Maharashtra, India) for storage. At regular intervals of 0 and 12 weeks, the liposomal dispersion was retrieved from storage and examined for characteristics such as creaming, color changes, and particle size. These evaluations help determine the stability and shelf life of the liposomal formulation under varying storage conditions [22]. 

### 2.5. In-Vitro Evaluation

#### 2.5.1. Cell Culture

MCF-7 cells (authentication No. 997/2021-22) were procured from the National Centre for Cell Sciences (NCCS), Pune, MH, India. The cells were confirmed for the absence of mycoplasma contamination and were tested for short tandem repeat (STR) analysis on sixteen STR loci. The MCF-7 cells were regularly cultured and maintained in DMEM supplemented with 10% heat-inactivated FBS, penicillin (100 units/mL), streptomycin (100 μg/mL), and gentamycin (100 μg/mL). This was performed in a CO_2_ incubator with a humidified environment at 37 °C and 5% CO_2_. 

#### 2.5.2. Cytotoxicity Assay

The cytotoxic potential of formulations was assessed by using the MTT assay. MCF-7 cells (1 × 10^5^) were seeded onto 96-well sterile plates and treated with variable concentrations of Placebo, PTX-Lipo, ALA-Lipo, and ALA-PTX-Lipo (1 μM, 2.5 μM, 5 μM, 7.5 μM, 10 μM, 15 μM, and 20 μM) for a duration of 24 h. Then, 15 μL of MTT (5 mg/mL) was added to the media, and the mixtures were incubated for 4 h at 37 °C in a CO_2_ incubator. DMSO (100 μL) was added, and after 10 min of incubation, cells were gently shaken to dissolve the blue-colored formazans crystal. The absorbance of the color solution was measured by using a microplate reader (Model 680 XR; Bio-Rad labs, Inc., Hercules, CA, USA) at a wavelength of 570 nm. The IC_50_ value and ½ IC_50_ value of PTX-Lipo, ALA-Lipo, and ALA-PTX-Lipo against MCF-7 cells were obtained by using a linear regression equation according to the curve-fitting method [23,24].

$$\%\;Cell\;Viability=\;\frac{Absorbance\;of\;treated\;cells-Absorbance\;of\;blank}{Absorbance\;of\;control\;cells-Absorbance\;of\;blank}\times 100$$

#### 2.5.3. Cellular Uptake by Confocal Microscopy

Cellular uptake was performed against MDA-MB-231 cells. Once the cells reached 90% confluence, the cell culture medium was aspirated, and the cells were washed three times with Hank’s Buffered Salt Solution (HBSS) from PAA Laboratories GmbH, Austria. A similar procedure was followed to prepare Coumarin-6-loaded liposome for cell uptake experiments for PTX-lipo. and ALA-PTX-Lipo. The cells were treated with Coumarin-6-loaded liposomes for 3 h and washed with HBSS (5 times). Following this, the cells were permeabilized by using 0.2% Triton (100×) and fixed with 3% paraformaldehyde from Merck, Karnataka, India. The cell nuclei were stained with DAPI from Sigma-Aldrich, USA, and the samples were examined by using an Olympus FV1000 confocal laser microscope (CLSM) for visualization and analysis [25,26]. 

#### 2.5.4. DAPI Staining

DAPI staining is a common method used to visualize chromatin fragmentation, particularly during processes like apoptosis. In our experiment with MCF-7 cells, they were seeded in 12-well plates. PTX-loaded liposomes (PTX-Lipo), ALA-loaded liposomes (ALA-Lipo), and ALA-PTX-loaded liposomes (ALA-PTX-Lipo) were treated with the IC50 dose for 24 h. After treatment, the cells were permeabilized with 0.1% Triton X-100 in PBS for 5 min, allowing the DAPI staining solution to penetrate the cells effectively. Subsequently, the cells were stained with 0.1% DAPI solution for 10 min to visualize the nuclei and any chromatin fragmentation that might occur. Finally, the stained cells were observed by using a fluorescent imaging device. Specifically a fluorescence microscope was used to examine the nuclear morphology and assess any apoptotic changes induced by the liposomal formulations (XL Olympus) [26]. 

#### 2.5.5. AO/EtBr Staining

AO/EtBr staining was used to observe apoptotic morphological changes. After 24 h of treatment with IC_50_ doses of Placebo, PTX-Lipo, ALA-Lipo, and ALA-PTX-Lipo on MCF-7 cells (1 × 10^5^), cells were examined with an inverted fluorescence microscope to look for morphological changes. Following separate harvesting, the untreated control cells and the PTX-Lipo-, ALA-Lipo-, and ALA-PTX Lipo-treated cells were rinsed with PBS and stained with AO (100 μg/mL) and EtBr (100 μg/mL) (1:1). The cells were promptly placed onto slides and examined by using an inverted fluorescence microscope (XL Olympus) to detect any alterations in the morphological characteristics associated with apoptosis [27].

$$\%\;of\;apoptotic\;cells=\frac{total\;number\;of\;apoptotic\;cells}{total\;number\;of\;cells\;counted}\times 100$$

#### 2.5.6. JC-1 Staining

Alteration in the mitochondrial membrane potential was analyzed by using the mitochondrial membrane potential-sensitive dye, JC-1. A stock solution of 1 mg/mL of JC-1 was prepared in 1% DMSO and stored at −20 °C shortly before use. After incubation for 24 h with the IC_50_ dose of untreated control cells, PTX-Lipo, ALA-Lipo, and ALA-PTX-Lipo cells were washed three times with PBS, and 200 μL of JC-1 (1 mg/mL) dye was added into each well. After incubation for 20 min, cells were washed three times with PBS, and images were taken by using an inverted fluorescence microscope (XL Olympus) [28]. 

#### 2.5.7. Statistical Analysis

The results were analyzed by using GraphPad Prism software (version 8.0.1). The findings are reported as means  ±  SD, with statistical significance evaluated by using one-way ANOVA followed by Dunnett’s test. Statistical significance was determined at * *p*  <  0.05, ** *p*  <  0.01, *** *p*  <  0.001, and **** *p*  <  0.0001 levels. 

## 3. Results

### 3.1. Pre-Formulation Study and DoE

The compatibility of PTX and ALA with the excipients (TPGS, PC, and CHO) was determined as color, odor, physical appearance, and melting point to avoid incompatibility (Table S1). Optimal conditions were determined by using the mathematical model developed from the Box–Behnken design, which describes the relationship between the input factors and the response variable(s). The model was then used to predict the optimal parameters, and validation experiments were performed to confirm these predictions. 

### 3.2. In- Vitro Characterization

#### 3.2.1. Particle Size and PDI, Zeta Potential, and Entrapment Efficiency

The optimized formulation was assessed for particle size, Polydispersity index (PDI), and Zeta potential (ZP) by using the Zeta sizer. The particle sizes of PTX-Lipo and ALA-PTX-Lipo were 162.80 ± 9.20 nm and 206.30 ± 9.96 nm, respectively. The PDIs of PTX-Lipo and ALA-PTX-Lipo were recorded to be 0.41 ± 0.05 and 0.35 ± 0.04. The entrapment efficiency (EE) was found to be 84% in PTX-Lipo and 80% in ALA-PTX Lipo. The results are summarized in Table 1. 

#### 3.2.2. FT-IR

**Figure 1:**
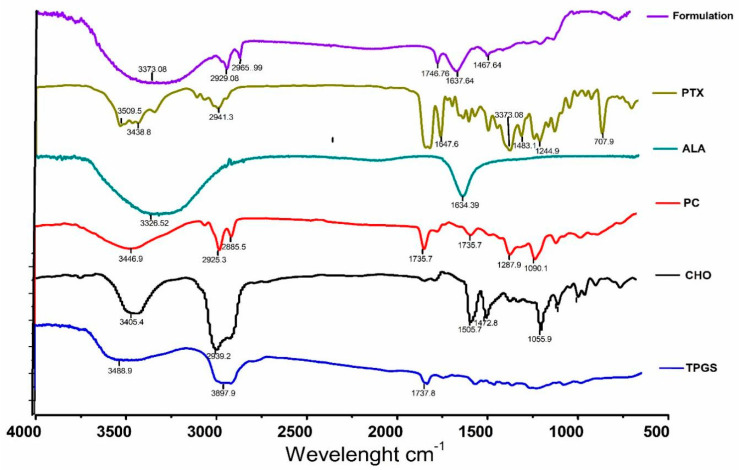
The compatibility of all compounds that were used for the development of liposomes by FTIR. The figure shows no major changes in peak shifting and functional groups.

Comparing the FTIR spectra of the physical mixtures of excipients to those of the individual excipients, pure drugs, and liposomes could not reveal any change in peaks. The observed peaks were the O-H stretching of alcohol from 32,550 to 3200, the C=O stretching of acid from 1750 to 17,335 of δ-lactone, and the C=C bending of alkenes group from 1648 to 1638 (Figure 1). 

#### 3.2.3. Surface Morphology

**Figure 2:**
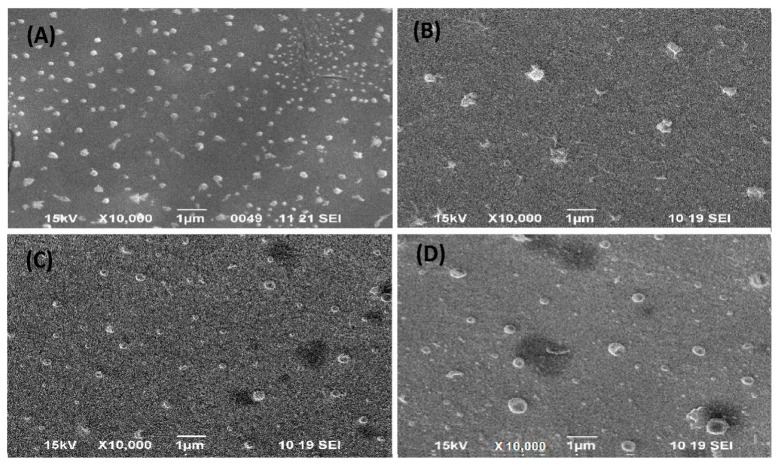
Surface morphology of (**A**) Placebo, (**B**) ALA-Liposome, (**C**) PTX-Liposome, and (**D**) ALA-PTX-Liposome determined by using scanning electron microscopy of liposomes.

**Figure 3:**
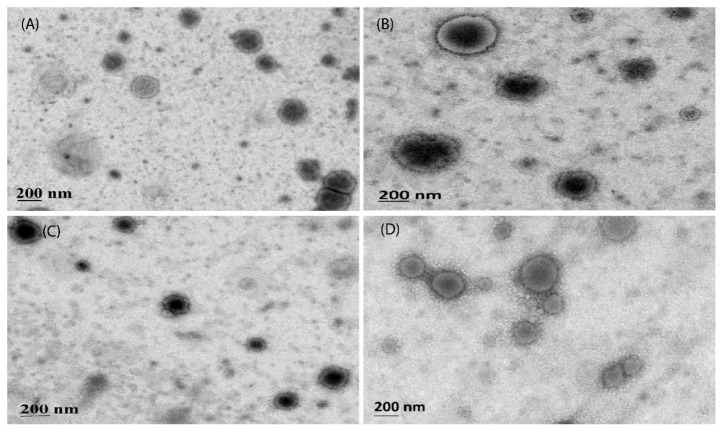
Surface morphology and internal structure of (**A**) Placebo, (**B**) ALA-Liposome, (**C**) PTX-Liposome, and (**D**) ALA-PTX-Liposome determined by using transmission electron microscopy of liposomes.

TEM and SEM confirmed the synthesized PTX-Lipo, ALA-Lipo, and ALA-PTX-Lipo, which exhibited separate and spherical structures. TEM analysis also revealed their internal morphology and affirmed a good correlation with particle size (Figure 2 and Figure 3). 

#### 3.2.4. In Vitro Drug Release

**Figure 4:**
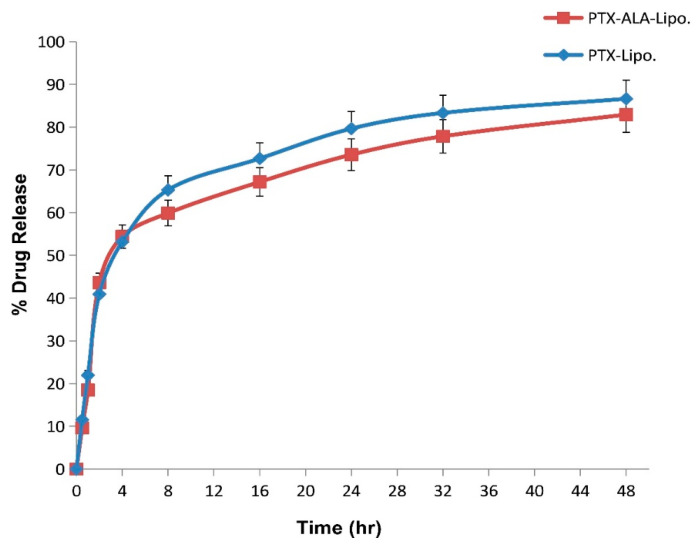
Pilot illustration of cumulative drug release profiles for PTX-Lipo and ALA-PTX-Lipo using the dialysis method in PBS (pH 7.4) at various time intervals up to 48 h.

All the liposomal formulations showed identical release profiles at pH 7.4 (PBS). Within 1 h, we recorded burst release values of 21.94% and 18.49% in PTX-Lipo and ALA-PTX-Lipo. After 16 h, we observed sustained release across all formulations, with 75% cumulative percentage of drug release. The early burst may have been due to drug leakage from the membrane of the liposomes (Figure 4). It was discovered that the correlation coefficient (R^2^) values of PTX-Lipo and ALA-PTX-Lipo for the zero order, first order, Higuchi model, and Korsmeyer–Peppas model were 0.659, 0.325, 0.931, and 0.751, and 0.642, 0.326, 0.932, and 0.924, respectively. According to these findings, the Higuchi kinetics model fits for PTX-Lipo and ALA-PTX-Lipo. The fitting of the drug release data to the Higuchi model indicates that diffusion is a significant mechanism in the drug release process. The model’s relatively high correlation suggests that drug release from the formulation is influenced by the square root of time, consistent with diffusion-controlled release. 

#### 3.2.5. Stability Study

The physical appearance of the liposomal formulations following the stability study is summarized in Table 2. The color of the preparations remained consistent over 12 weeks of storage. No sedimentation was observed after 12 weeks at either 8 °C or 37 °C. The particle size and Zeta potential of PTX-Lipo and ALA-PTX-Lipo were measured at 8 °C after 12 weeks, with values of 215.6 ± 16.7 nm and −18.9 ± 3.7 mV for PTX-Lipo and 288.4 ± 57 nm and −16.2 ± 1.9 mV for ALA-PTX-Lipo. At 37 °C, the measurements were 227.8 ± 32 nm and −21.2 ± 7.5 mV for PTX-Lipo, and 317.6 ± 40 nm and −19.2 ± 6.3 mV for ALA-PTX-Lipo. Based on these findings, both PTX-Lipo and ALA-PTX-Lipo were stable and suitable for storage for 12 weeks at both 8 °C and 37 °C (Table 2). 

### 3.3. In Vitro Cell Culture Study

#### 3.3.1. Cytotoxicity Assay

**Figure 5:**
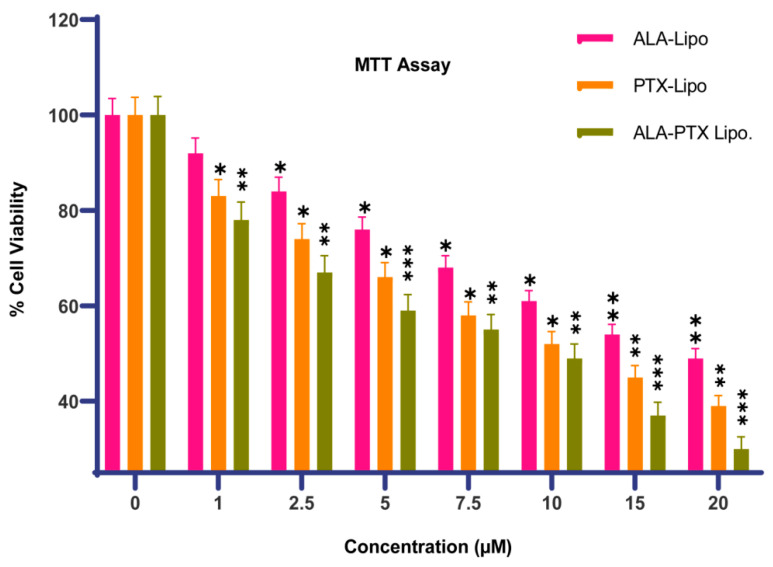
The figure demonstrates the percentage of cell viability after 24 h of incubation at half the IC50. Statistical significance was determined at * *p*  <  0.05, ** *p*  <  0.01, *** *p*  <  0.001, and comparisons were made with the control.

Significant growth inhibition of MCF-7 cells was observed in response to PTX-Lipo, ALA-Lipo, and ALA-PTX-Lipo at concentrations of 1–20 nM, in a dose-dependent manner. The IC50 values for PTX-Lipo, ALA-Lipo, and ALA-PTX-Lipo against MCF-7 cells were 11.51 µM, 12.56 µM, and 10.61 µM, respectively (Figure 5). 

#### 3.3.2. Cellular Uptake

**Figure 6:**
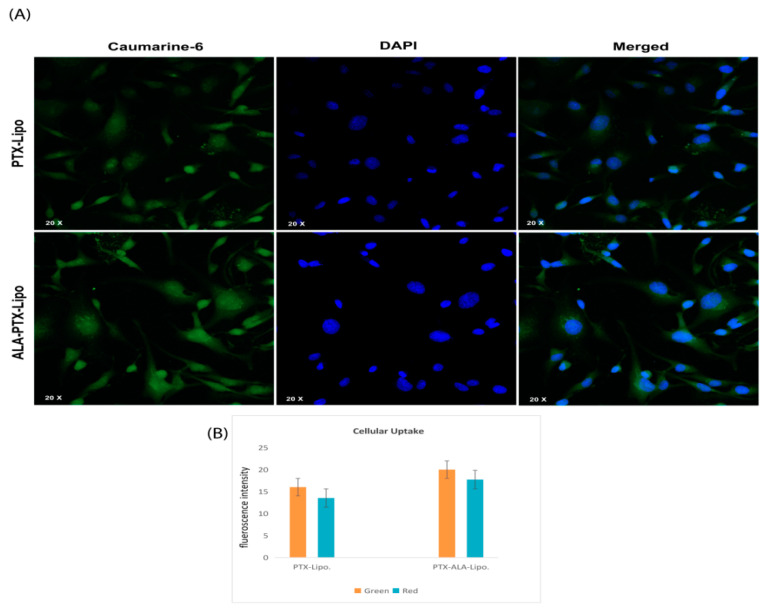
(**A**) Internalization and distribution of liposomes in cells. Caumarine-6 dye was internalized into the cytoplasm (green), and DAPI staining (blue) labelled the nucleus of the cell. The merged panel indicates the internalization of liposomes into both the cytoplasm and the nucleus(magnification 20×). (**B**) Plot showing the ALA-Lipo and ALA-PTX-Lipo cell uptake comparison in quantitative data.

The degree of internalization and the intracellular distribution of the liposomes in MDA-MB-231 cells were observed by using fluorescence microscopy. Images were captured by using the DAPI channel (blue), the FITC channel (green), and an overlay of the two channels to assess the colocalization and distribution of the liposomes within the cells (Figure 6). 

#### 3.3.3. DAPI Staining

**Figure 7:**
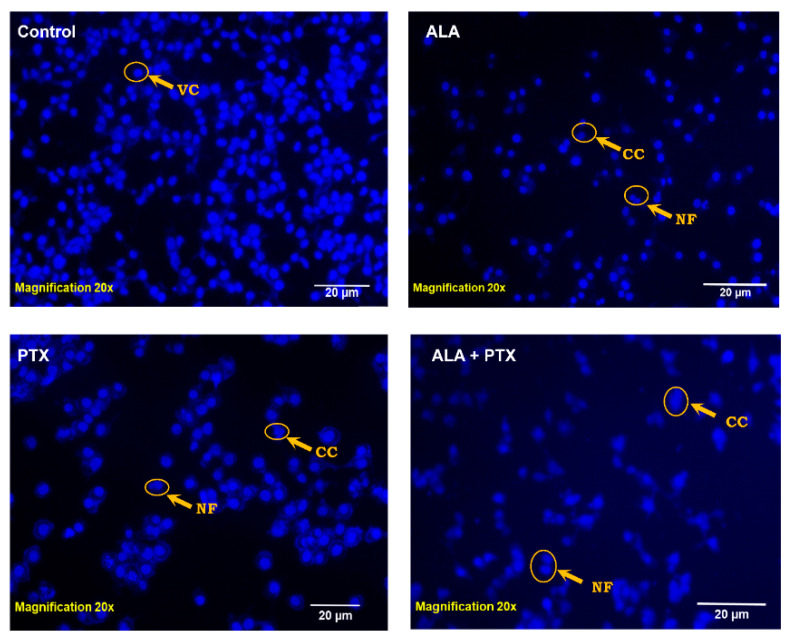
The DAPI-stained nuclei of MCF-7 cells are shown in the figure with changes in their morphology. Furthermore, DAPI staining following treatment with PTX-Lipo, ALA-Lipo, and ALA-PTX-Lipo demonstrated VC (viable cells), CC (chromatin condensation), and NF (nuclear fragmentation), indicated by an arrow, in comparison to the control group.

DAPI is a prominent nuclear counterstain, and DAPI staining revealed that treatment with PTX-Lipo, ALA-Lipo, and ALA-PTX-Lipo induced nuclear fragmentation in MCF-7 cells. Untreated cells displayed normal, intact nuclei. After treatment with PTX-Lipo, ALA-Lipo, and ALA-PTX-Lipo, apoptotic nuclei with condensed or fragmented chromatin were observed. The nuclear morphology studies showed classic apoptotic changes such as chromatin condensation, nuclear fragmentation, and the formation of apoptotic bodies in MCF-7 cells. Among the formulations, ALA-PTX-Lipo demonstrated significant apoptotic markers compared with PTX-Lipo and ALA-Lipo (Figure 7). 

#### 3.3.4. AO/EtBr Staining

**Figure 8:**
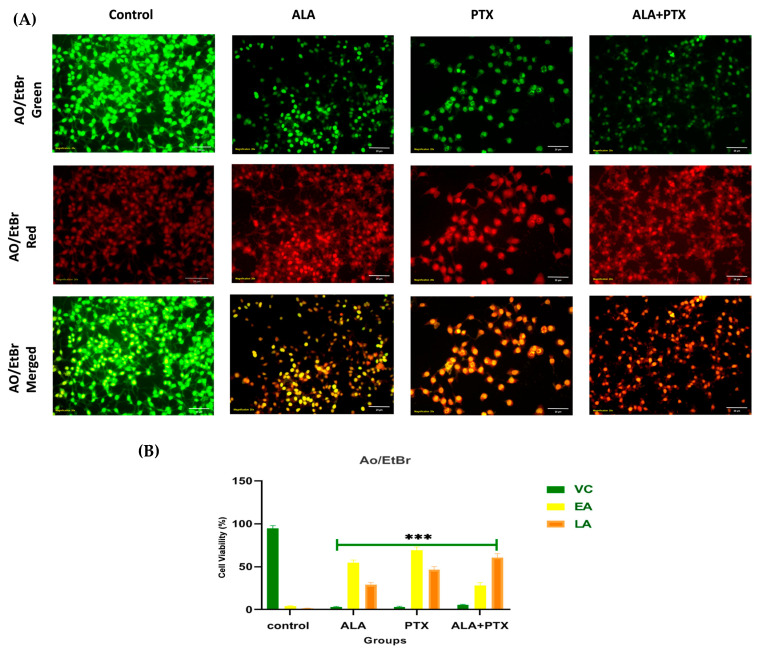
(**A**) The figure illustrates apoptotic morphological changes in MCF-7 cells stained with AO/EtBr. Additionally, treatment with PTX-Lipo, ALA-Lipo, and ALA-PTX-Lipo in AO/EtBr staining revealed early apoptotic regions marked by yellow areas and late apoptotic nuclei appearing bright orange compared with the control group. (**B**) The quantitative data also showed notable variations among groups. VC: viable cells; LA: late apoptosis. An inverted fluorescent microscope with 20× magnification was used for capturing the pictures. Statistical significance was determined at *** *p*  <  0.001, and comparisons were made with the control.

AO/EtBr staining validated the early and late apoptotic changes in ER + MCF-7 cells caused by PTX-Lipo, ALA-Lipo, and ALA-PTX-Lipo with fluorescence microscopy. Staining with AO/EtBr revealed the presence of apoptotic markers such as chromatin condensation, apoptotic body formation, membrane blebbing, and fragmented nuclei in ER + MCF-7 cells (Figure 8). 

#### 3.3.5. JC-1 Staining

**Figure 9:**
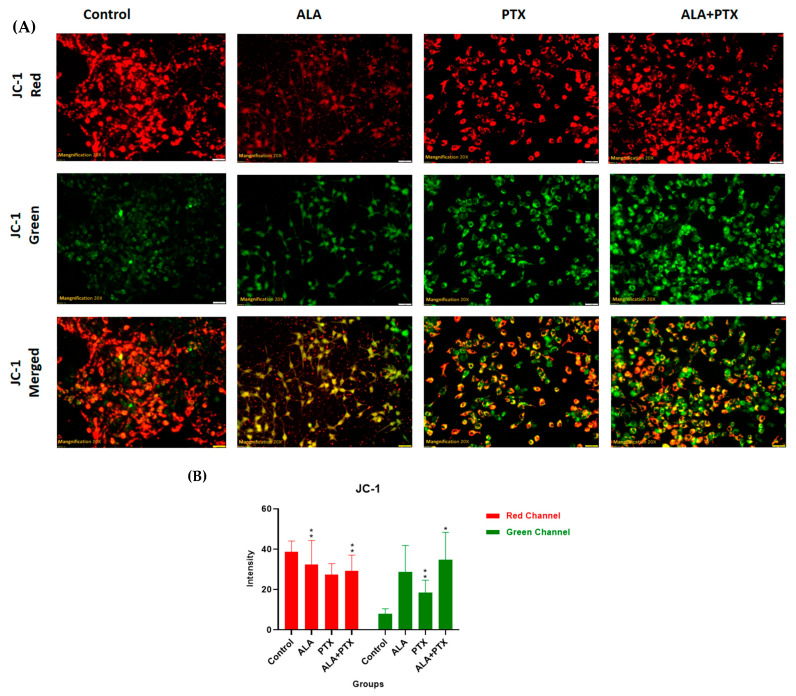
(**A**) JC-1 staining was performed on MCF-7 cells to evaluate mitochondrial membrane potential and pictures were taken with 20× magnification. Treatment with PTX-Lipo, ALA-Lipo, and ALA-PTX-Lipo increased green fluorescence of the JC-1 monomer, indicating a lower mitochondrial potential compared with the control group, which exhibited red fluorescence indicative of high mitochondrial potential. (**B**) The qualitative data also showed notable variation among the groups. Statistical significance was determined at * *p* < 0.05, ** *p* < 0.01, and comparisons were made with the control.

Mitochondrial membrane potential was assessed by using the voltage-sensitive cationic dye JC-1. In untreated cells, red fluorescence was observed through fluorescence microscopy, indicating the presence of the J-aggregate dimeric form of JC-1. Treatment with IC_50_ concentrations of PTX-Lipo, ALA-Lipo, and ALA-PTX-Lipo enhanced the absorption of JC-1 in cells, indicating apoptosis and mitochondrial membrane depolarization (Figure 9). 

## 4. Discussion

The pursuit of breakthroughs in anticancer therapy remains critical to reducing cancer-related deaths. Nanotechnology, with its rapid advancements in medical science, offers promising solutions to longstanding clinical challenges. Various unique nanoparticles have been developed, each with distinct features, such as particle size, shape, charge, surface modification, and therapeutic impact [29]. Breast cancer and many other tumors are commonly treated by using a combination chemotherapeutic approach that integrates biological drugs, leveraging synergistic or additive effects through diverse mechanisms [30]. Paclitaxel (PTX), a taxane, stands out as an effective anti-neoplastic agent that impacts microtubule dynamics, leading to mitotic arrest and cell death [31]. However, PTX is associated with increased risks of hypersensitivity reactions, neuropathy, and challenges in drug delivery to tumors, leading to higher toxicity and reduced therapeutic efficacy [32,33]. Previous studies reported on PTX carried by drug delivery system to enhance anticancer efficacy and reduce drug-related side effects [34,35]. Previous studies have reported that polyunsaturated fatty acids (PUFAs) like arachidonic acid (AA) are vital components of cell membranes, regulating membrane fluidity and various cell signaling pathways, including inflammation. ALA, an essential omega-3 fatty acid, competes with AA physiologically, offering potential therapeutic benefits [10]. Hence, this study aimed to develop ALA-based liposomes encapsulating PTX to enhance cellular uptake, reduce therapeutic doses, and mitigate PTX-related side effects, leveraging the synergistic potential of ALA and PTX. The physical compatibility of excipients and liposomes was confirmed through FTIR analysis, showing no significant changes in peak patterns, affirming the suitability of chosen components for further development. BBD optimization of liposomal formulation replaced 90% of phosphatidylcholine (PC) with ALA in liposomes, followed by PTX loading. The optimized PTX-Lipo and ALA-PTX-Lipo exhibited satisfactory particle size, PDI, and drug entrapment efficiency. The in vitro drug release studies showed initial burst release followed by sustained release in both PTX-Lipo and ALA-PTX-Lipo, with ALA-PTX-Lipo exhibiting sustained release patterns up to 48 h. and following Higuchi fitted model kinetics. The morphological analyses confirmed the spherical unilamellar structure of the liposomes. The cellular uptake studies demonstrated significant uptake of all formulations, with slightly higher uptake observed for ALA-based liposomes, attributed to increased fluidity conferred by ALA. The accelerated stability studies indicated stability over 12 weeks, with a slight increase in size observed for all formulations. The anticancer efficacy studies in MCF-7 cells showed concentration-dependent reductions in cell viability, with ALA-PTX-Lipo demonstrating the lowest IC_50_ value, indicating enhanced cytotoxicity compared with the other formulations. The morphological analysis revealed apoptotic changes in cells treated with all formulations, with ALA-PTX-Lipo inducing more pronounced changes, indicating enhanced anticancer efficacy. Further investigations using AO/EtBr and DAPI staining confirmed apoptotic alterations in cells treated with liposomal formulations, with ALA-PTX-Lipo showing more significant apoptotic changes. JC-1 staining revealed increased green fluorescence and decreased red fluorescence (decreased red/green ratio) in cells treated with ALA-PTX-Lipo, indicating mitochondrial membrane depolarization, a hallmark of early apoptosis, confirming the enhanced cytotoxic efficacy of the formulation. In summary, ALA-PTX-Lipo demonstrated superior anticancer efficacy compared with PTX-Lipo and ALA-Lipo, highlighting the potential of ALA to enhance the therapeutic outcomes of PTX in cancer treatment. 

## 5. Conclusions

In conclusion, our study utilizes ALA-based liposomes containing PTX to improve therapeutic outcomes while minimizing side effects. We employed a systematic approach, including compatibility assessments, optimization using the Box–Behnken design, and comprehensive characterization, to develop ALA-PTX liposomes. Despite a slight increase in particle size and a minor decrease in drug entrapment efficiency due to ALA integration, these liposomes exhibited comparable drug release profiles to PTX liposomes, maintaining sustained release over 48 h. Cellular uptake studies indicated enhanced uptake of ALA-PTX liposomes compared with PTX liposomes, likely attributed to ALA’s enhanced fluidity. Moreover, the liposomes demonstrated stability over 12 weeks. ALA-PTX liposomes demonstrated superior efficacy against MCF-7 cells, inducing pronounced apoptotic changes. The enhanced therapeutic efficacy of the combination highlighted the potential of ALA-based liposomes as an effective delivery system for improving PTX delivery and potentially other anticancer medications. However, further evaluations regarding the toxicity, pharmacokinetics, and pharmacodynamics of ALA-PTX are warranted. 

## Figures and Tables

**Table 1:** Characterization of optimized liposome for particle size, particle distribution index, Zeta potential, and entrapment efficiency.

Formulation	Particle Size (nm)	PDI	Zeta Potential (mV)	Entrapment Efficiency (%)
Placebo	133.43 ± 3.95	0.39 ± 0.02	−15.00 ± 5.76	-
PTX-lipo	162.80 ± 9.20	0.41 ± 0.05	−22.40 ± 4.80	84.70 ± 0.05
ALA-lipo	154.67 ± 8.70	0.32 ± 0.02	−22.00 ± 7.80	-
ALA-PTX-lipo	206.30 ± 9.96	0.35 ± 0.04	−20.80 ± 4.34	80.58 ± 0.07

Values are presented as means ± SD (*n* = 3).

**Table 2:** Data depicting the accelerated stability study of ALA-Lipo, PTX-Lipo, and ALA-PTA-Lipo following the storage at 8 °C and 37 °C for 12 weeks.

Liposome	Initial		After 12 Weeks			
	PS (nm)	ZP	8 °C		37 °C	
			PS (nm)	ZP (mV)	PS (nm)	ZP (mV)
ALA-Lipo	154.7 ± 8.7	−22.0 ± 4.8	200.6 ± 6.3	−20.4 ± 5.3	187.2 ± 5.8	−16.8 ± 3.7
PTX- Lipo	162.5 ± 9.2	−22.4 ± 7.8	215.6 ± 16.7	−18.9 ± 3.7	227.8 ± 3.2	−21.2 ± 7.5
ALA-PTA-Lipo	206.0 ± 9.9	−20.8 ± 4.3	288.4 ± 15.2	−16.2 ± 1.9	317.6 ± 4.0	−19.2 ± 6.3

Values are presented as means ± SD (*n* = 3).

## Data Availability

Data is contained within the article and supplementary material.

## Supplementary Materials

The following supporting information can be downloaded at: https://www.mdpi.com/article/10.3390/pharmaceutics16070913/s1. Figure S1: A 3D surface plot illustrating the inter-relationship between dependent variables, including Phosphatidylcholine (PC), Cholesterol (CHO), and α-linolenic acid (ALA), and independent variables such as Particle size, Particle size distribution (PDI), and Zeta potential. The plot is segmented into three sections: (I) Particle size, (II) PDI, and (III) Zeta potential, each showcasing the relationships between the mentioned variables in a three-dimensional space. Table S1: Preformulation data of PTX and ALA. Table S2: Data depicting the values of dependent and independent variables used for the design of the experiment (DoE). Table S3:Design of Experiments (DoE) for optimization of liposome.

## References

[1] Chavez J.D., Keller A., Zhou B., Tian R., Bruce J.E. (2019). Cellular interactome dynamics during paclitaxel treatment. Cell Rep..

[2] Iacoviello L., Bonaccio M., de Gaetano G., Donati M.B. (2021). Epidemiology of breast cancer, a paradigm of the “common soil” hypothesis. Semin. Cancer Biol..

[3] Narmani A., Jafari S.M. (2021). Chitosan-based nanodelivery systems for cancer therapy: Recent advances. Carbohydr. Polym..

[4] Mansara P.P., Deshpande R.A., Vaidya M.M., Kaul-Ghanekar R. (2015). Differential ratios of omega fatty acids (AA/EPA+ DHA) modulate growth, lipid peroxidation and expression of tumor regulatory MARBPs in breast cancer cell lines MCF7 and MDA-MB-231. PLoS ONE.

[5] Zalba S., Ten Hagen T.L. (2017). Cell membrane modulation as adjuvant in cancer therapy. Cancer Treat. Rev..

[6] Szlasa W., Zendran I., Zalesińska A., Tarek M., Kulbacka J. (2020). Lipid composition of the cancer cell membrane. J. Bioenerg. Biomembr..

[7] Mathew S.A., Bhonde R.R. (2018). Omega-3 polyunsaturated fatty acids promote angiogenesis in placenta derived mesenchymal stromal cells. Pharmacol. Res..

[8] Das U.N. (2021). “Cell membrane theory of senescence” and the role of bioactive lipids in aging, and aging associated diseases and their therapeutic implications. Biomolecules.

[9] Singla A.K., Garg A., Aggarwal D. (2002). Paclitaxel and its formulations. Int. J. Pharm..

[10] Yadav R.K., Singh M., Roy S., Ansari M.N., Saeedan A.S., Kaithwas G. (2018). Modulation of oxidative stress response by flaxseed oil: Role of lipid peroxidation and underlying mechanisms. Prostaglandins Other Lipid Mediat..

[11] Singh M., Kaithwas G. (2017). Alpha-Linolenic Acid Mediated Stabilization of Hif-1-Alpha and Downregulation Fasn to Inhibit Palmitic Acid Synthesis and Activation of Mitochondrial Apoptosis for Mammary Gland Chemoprevention. J. Cancer Res. Ther..

[12] Weissman S.A., Anderson N.G. (2015). Design of experiments (DoE) and process optimization. A review of recent publications. Org. Process Res. Dev..

[13] Kushwah V., Jain D.K., Agrawal A.K., Jain S. (2018). Improved antitumor efficacy and reduced toxicity of docetaxel using anacardic acid functionalized stealth liposomes. Colloids Surf. B Biointerfaces.

[14] Jurić Simčić A., Erak I., Cetina Čižmek B., Hafner A., Filipović-Grčić J. (2023). Selection of Excipients for the Preparation of Vancomycin-Loaded Poly (D, L-lactide-co-glycolide) Microparticles with Extended Release by Emulsion Spray Drying. Pharmaceutics.

[15] Ling Y., Huang Y. Preparation and release efficiency of poly (lactic-co-glycolic) acid nanoparticles for drug loaded paclitaxel. Proceedings of the 7th Asian-Pacific Conference on Medical and Biological Engineering: APCMBE 2008.

[16] Liu Y., Liu D., Zhu L., Gan Q., Le X. (2015). Temperature-dependent structure stability and in vitro release of chitosan-coated curcumin liposome. Food Res. Int..

[17] Jeon S., Yoo C.Y., Park S.N. (2015). Improved stability and skin permeability of sodium hyaluronate-chitosan multilayered liposomes by Layer-by-Layer electrostatic deposition for quercetin delivery. Colloids Surf. B Biointerfaces.

[18] Li R., Deng L., Cai Z., Zhang S., Wang K., Li L., Ding S., Zhou C. (2017). Liposomes coated with thiolated chitosan as drug carriers of curcumin. Mater. Sci. Eng. C.

[19] Dave V., Gupta A., Singh P., Gupta C., Sadhu V., Reddy K.R. (2019). Synthesis and characterization of celecoxib loaded PEGylated liposome nanoparticles for biomedical applications. Nano-Struct. Nano-Objects.

[20] Perrie Y., Mohammed A.U., Vangala A., McNeil S.E. (2007). Environmental scanning electron microscopy offers real-time morphological analysis of liposomes and niosomes. J. Liposome Res..

[21] Wang Z.-Y., Zhang H., Yang Y., Xie X.-Y., Yang Y.-F., Li Z., Li Y., Gong W., Yu F.-L., Yang Z. (2016). Preparation, characterization, and efficacy of thermosensitive liposomes containing paclitaxel. Drug Deliv..

[22] Bhalerao S., Raje Harshal A. (2003). Preparation, optimization, characterization, and stability studies of salicylic acid liposomes. Drug Dev. Ind. Pharm..

[23] Arunasree K.M. (2010). Anti-proliferative effects of carvacrol on a human metastatic breast cancer cell line, MDA-MB 231. Phytomedicine.

[24] Gupta U., Sharma S., Khan I., Gothwal A., Sharma A.K., Singh Y., Chourasia M.K., Kumar V. (2017). Enhanced apoptotic and anticancer potential of paclitaxel loaded biodegradable nanoparticles based on chitosan. Int. J. Biol. Macromol..

[25] de Castro C.E., Ribeiro C.A., Alavarse A.C., Albuquerque L.J., da Silva M.C., Jäger E., Surman F., Schmidt V., Giacomelli C., Giacomelli F.C. (2018). Nanoparticle–cell interactions: Surface chemistry effects on the cellular uptake of biocompatible block copolymer assemblies. Langmuir.

[26] Suganya K.S.U., Govindaraju K., Prabhu D., Arulvasu C., Karthick V., Changmai N.J.A.S.S. (2016). Anti-proliferative effect of biogenic gold nanoparticles against breast cancer cell lines (MDA-MB-231 & MCF-7). Appl. Surf. Sci..

[27] Rastogi S., Ansari M.N., Saeedan A.S., Singh S.K., Mukerjee A., Kaithwas G. (2024). Novel furan chalcone modulates PHD-2 induction to impart antineoplastic effect in mammary gland carcinoma. J. Biochem. Mol. Toxicol..

[28] Roy S., Singh M., Rawat A., Kumar D., Kaithwas G. (2020). Mitochondrial apoptosis and curtailment of hypoxia-inducible factor-1α/fatty acid synthase: A dual edge perspective of gamma linolenic acid in ER+ mammary gland cancer. Cell Biochem. Funct..

[29] Tanaka T., Decuzzi P., Cristofanilli M., Sakamoto J.H., Tasciotti E., Robertson F.M., Ferrari M. (2009). Nanotechnology for breast cancer therapy. Biomed. Microdevices.

[30] Von Hoff D.D., Layard M.W., Basa P., Davis H.L., Von Hoff A.L., Rozencweig M., Muggia F.M. (1979). Risk factors for doxorubicin-lnduced congestive heart failure. Ann. Intern. Med..

[31] Bernabeu E., Cagel M., Lagomarsino E., Moretton M., Chiappetta D.A. (2017). Paclitaxel: What has been done and the challenges remain ahead. Int. J. Pharm..

[32] Ten Tije A.J., Verweij J., Loos W.J., Sparreboom A. (2003). Pharmacological effects of formulation vehicles: Implications for cancer chemotherapy. Clin. Pharmacokinet..

[33] Sparreboom A., Van Zuylen L., Brouwer E., Loos W.J., De Bruijn P., Gelderblom H., Pillay M., Nooter K., Stoter G., Verweij J. (1999). Cremophor EL-mediated alteration of paclitaxel distribution in human blood: Clinical pharmacokinetic implications. Cancer Res..

[34] Xu M.-Q., Hao Y.-L., Wang J.-R., Li Z.-Y., Li H., Feng Z.-H., Wang H., Wang J.-W., Zhang X. (2021). Antitumor activity of α-linolenic acid-paclitaxel conjugate nanoparticles: In vitro and in vivo. Int. J. Nanomed..

[35] Gu Q., Xing J.Z., Huang M., Zhang X., Chen J. (2013). Nanoformulation of paclitaxel to enhance cancer therapy. J. Biomater. Appl..

