# Correction: Kumar et al. Harnessing Potential of ω-3 Polyunsaturated Fatty Acid with Nanotechnology for Enhanced Breast Cancer Therapy: A Comprehensive Investigation into ALA-Based Liposomal PTX Delivery. *Pharmaceutics* 2024, *16*, 913

**DOI:** 10.3390/pharmaceutics18040479

**Published:** 2026-04-14

**Authors:** Rohit Kumar, Anurag Kumar, Dharmendra Kumar, Sneha Yadav, Neeraj Kumar Shrivastava, Jyoti Singh, Archana Bharti Sonkar, Pratibha Verma, Dilip Kumar Arya, Gaurav Kaithwas, Ashish Kumar Agrawal, Sanjay Singh

**Affiliations:** 1 Department of Pharmaceutical Sciences, School of Pharmaceutical Sciences, Babasaheb Bhimrao Ambedkar University (A Central University), Vidya Vihar, Raebareli Road, Lucknow 226025, India; rohitpharm06@gmail.com (R.K.); anuragkumar173@gmail.com (A.K.); dkpharm.25@hotmail.com (D.K.); sneha1991yad@gmail.com (S.Y.); neerajsrivastava91@gmail.com (N.K.S.); jsjyoti156@gmail.com (J.S.); sonkararchana818283@gmail.com (A.B.S.); pratibhaverma2208@gmail.com (P.V.); dkaharsh101@gmail.com (D.K.A.); gauravk@bbau.ac.in (G.K.); 2 Department of Pharmaceutical Engineering and Technology, Indian Institute of Technology (BHU), Varanasi 221005, India; 3 Dr. Shakuntala Misra National Rehabilitation University, Mohaan Road, Lucknow 226017, India

**Text Correction**

There was an error in the original publication [1]. The statement in Section 4 (Discussion) that reads, “JC-1 staining revealed increased red fluorescence, indicating higher mitochondrial membrane potential in cells treated with ALA-PTX-Lipo, suggesting improved cytotoxicity”, contains a typographical error.

The corrected statement in Section 4 is as follows:

“JC-1 staining revealed increased green fluorescence and decreased red fluorescence (decreased red/green ratio) in cells treated with ALA-PTX-Lipo, indicating mitochondrial membrane depolarization, a hallmark of early apoptosis, confirming the enhanced cytotoxic efficacy of the formulation.”

In the published article [1], clarification is required regarding the selection of the optimal liposomal formulation obtained from the Box–Behnken design and its relationship to the final formulation used in subsequent experiments.

The authors acknowledge that the description of the optimization process lacked sufficient clarity. Specifically, a discrepancy exists between the optimized formulation parameters reported from the Box–Behnken design and the formulation ultimately selected for experimental evaluation.

To clarify the methodology, the following statement has been added to Section 2. Material and Methods, Section 2.3. Liposome Preparation and Optimization, Subsection 2.3.1. Design of Experiment:

“Using Design Expert software with the Box-Behnken design, the optimal formulation was determined using desirability function analysis targeting minimized particle size (<200 nm), optimized PDI (<0.4), and appropriate zeta potential for stability. The predicted optimal parameters were PC: 15 mg, CHOL: 10 mg, and ALA: 55 μL, yielding predicted responses of particle size 154.4 nm, PDI 0.25, and zeta potential −22 mV. The final experimental formulation used PC: 20 mg, CHOL: 10 mg, ALA: 60 μL, which was based on the maximum entrapment and loading efficiency observed with this ratio as compared to the optimized formulation.”

The authors state that the scientific conclusions are unaffected. This correction was approved by the Academic Editor. The original publication has also been updated.
