# Supplementary material for: Harnessing Potential of ω-3 Polyunsaturated Fatty Acid with Nanotechnology for Enhanced Breast Cancer Therapy: A Comprehensive Investigation into ALA-Based Liposomal PTX Delivery
Source: Pharmaceutics. 2024 Jul 9;16(7):913. doi: 10.3390/pharmaceutics16070913 (PMC11279858; doi:10.3390/pharmaceutics16070913)
Supplement: Supplementary file 1 [file pharmaceutics-16-00913-s001.zip › pharmaceutics-3063003-supplementary.pdf]

Supplementary Materials

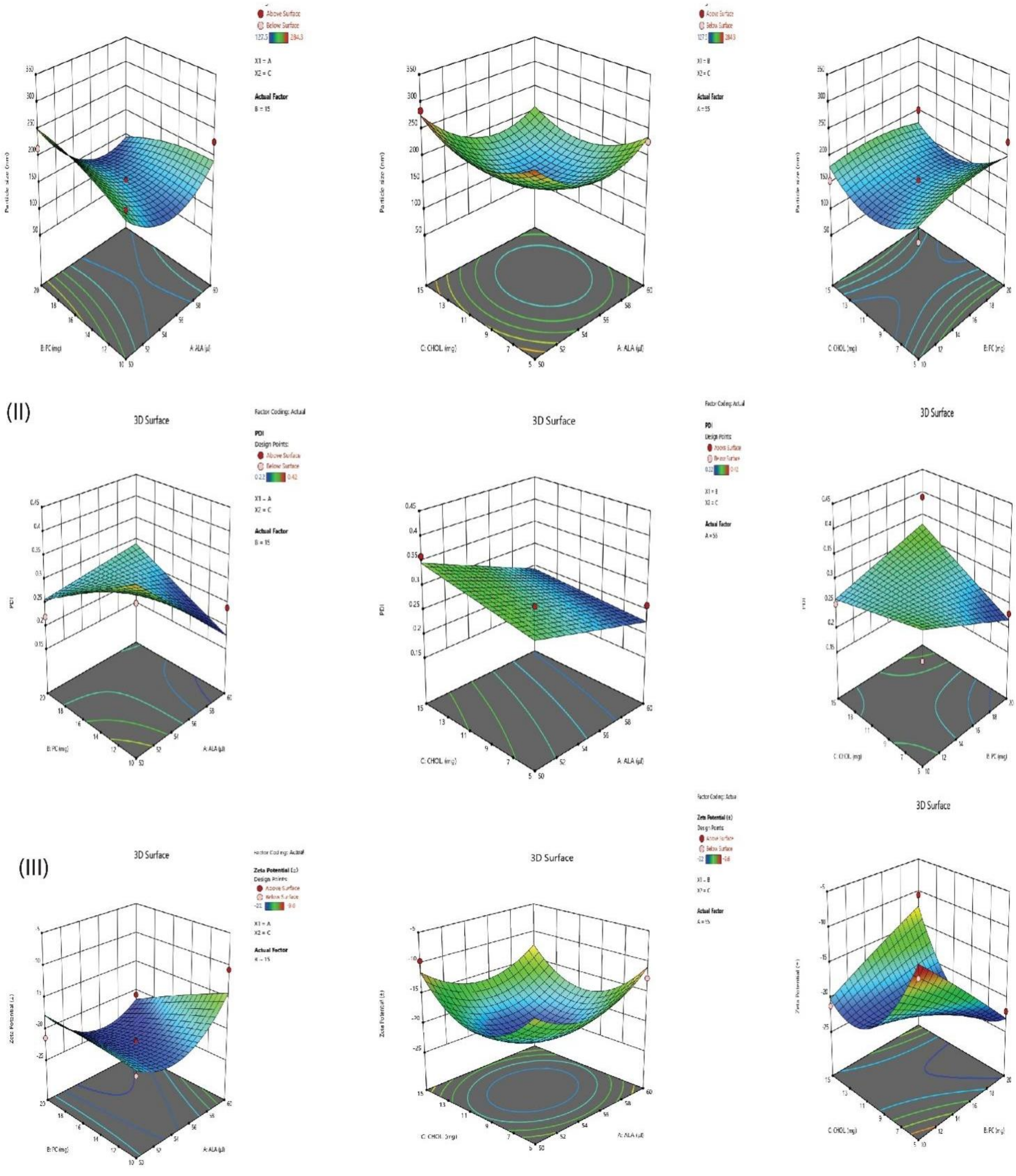

Figure S1: A 3D surface plot illustrating the inter-relationship between dependent variables, including Phosphatidylcholine (PC), Cholesterol (CHO), and α-linolenic acid (ALA), and independent variables such as Particle size, Particle size distribution (PDI), and Zeta potential. The plot is segmented into three sections: (I) Particle size, (II) PDI, and (III) Zeta potential, each showcasing the relationships between the mentioned variables in a three-dimensional space

Table S1: Preformulation data of PTX and ALA

Parameter	PTX		ALA	
	**Observed Value**	**Literature Value**	**Observed Value**	**Literature Value**
Physical appearance/color	White fine powder	White fine powder	Yellowish liquid	Yellowish liquid
Odor	Odorless	Odorless	Low odor	Low odor
Melting Point	220°C	213-220°C	-	-
λmax	230nm	227-230nm	-	-

PTX: Paclitaxel, ALA: Alpha Linolenic acid

Table S2: Data depicting the values of dependent and independent variables used for the design of the experiment (DoE).

**INDEPENDENT VARIABLES**		**Low**	**Medium**	**High**
X_1	PC (mg)	10	15	20
X_2	CH (mg)	05	10	15
X_3	ALA (µL)	50	55	60
**DEPENDENT VARIABLES:** Y_1:Particle size, Y_2:Particle size distribution, Y_3: Zeta potential				

ALA: Alpha linolenic acid, PC:Phosphotidycholine, CH: Cholesterol

Table S3: Design of Experiments (DoE) for optimization of liposome, The initial step involved selecting a candidate model based on theoretical considerations and prior empirical evidence. The parameters of this model were then estimated using standard fitting techniques. The goodness of fit was measured by the R^2 value, which provided a measure of how well the model explained the variability of the response variable. A higher R^2 indicated a better fit. Additionally, the adjusted R^2 value was measured to account for the number of predictors in the model, providing a more accurate assessment of model performance in the context of multiple predictors. The root mean square error (RMSE) was also used to quantify the differences between observed and predicted values. Model diagnostics were assessed to ensure the validity and reliability of the model. Thus, the software identified the optimal combination of the independent variables that resulted in the desired characteristics of the liposomes, such as smaller PS, lower PDI, and appropriate ZP values. the optimal formulation (highlighted red) was achieved through a systematic approach involving multiple stages of analysis and evaluation.

**F-1 PC(mg)**	**F-2 CHO (mg)**	**F-3 ALA (µL)**	**R-1 Particle Size(nm)**	**R-2, PDI**	**R-3, Zeta Potential(mV)**
20	5	55	227	0.23	-22
15	5	50	284.3	0.36	-9.6
15	10	55	154.4	0.25	-22
20	10	50	215	0.22	-21.4
15	5	60	227.3	0.26	-12.7
15	10	55	154.4	0.25	-22
10	15	55	154.4	0.25	-21.2
20	15	55	202.3	0.39	-10.5
15	15	50	284.3	0.36	-9.6
10	10	60	227.2	0.24	-10.6
10	10	50	208.8	0.42	-19.5
15	10	55	154.4	0.25	-22
20	10	60	206.5	0.35	-20.8
15	10	55	154.4	0.25	-22
15	10	55	154.4	0.25	-22
15	15	60	200.2	0.23	-15
10	5	55	154.4	0.25	-10.5

ALA: α- Linolenic acid, PC: Phosphotidylcholine, CHO: Cholesterol, PDI: Particle sizedistribution
